# Extra Supplement—The Nursing Mirror

**Published:** 1889-04-20

**Authors:** 


### The Nursing Mirror.

All Contributions for this Supplement should be addressed "The Nursing Mirror," care of the Editor, 139-140, Salisbury Court, Fleet Street, London, E.C.

### En Passant.

A HINT FOR EVERYONE.—A short time ago an inquest was held on the body of a boy, aged five weeks, who died suddenly from spasm of the glottis. At the enquiry it was stated that if a mother or anyone having a child seized with spasm of the throat would insert a finger and open the glottis, breathing would be resumed, and the life saved.

DISTRICT NURSES AT BRISTOL. — The annual meeting of the Bristol District Nurses' Society took place in the beginning of the month, and a special appeal for funds was made. The receipts last year were £941, and the expenses £935. An auxiliary nurse has been appointed, who will be ready to take temporary work for a nurse when disabled, or to undertake night nursing in serious cases. This appointment meets a want long felt by the society, but is, of course, a great extra expense. There are still many districts unsupplied with a nurse owing to lack of funds. The Rev. J. R. Graham, of St. Jude's, in referring to this, said that indiscriminate almsgiving was doing an immense injury to the moral welfare of the Bristol poor. It was stated that the sum of £50,000 a year was given in private alms by the inhabitants of Clifton and Redland, and he was afraid that a large proportion of that money went to the idle, the worthless, and the undeserving. Let that money rather go through some recognised channel, and there was none better than the Bristol District Nurses' Society.

NURSING LECTURES AT PORTSMOUTH.—A series of lectures on nursing are being given at the Portsmouth Hospital, Dr. J. Ward Cousins inaugurating the series by an interesting address. He traced the history of nursing from the period of the Compassionate Brothers, who were founded in Spain in 1540, and showed that out of this institution sprang the Compassionate Sisters in 1655. Both institutions received Papal sanction, but in the numerous wars that subsequently arose the societies became extinct, though they were revived in 1807 by the great Napoleon. Dr. Cousins next dealt with the scope for the study of the science in that hospital, and described the course pursued by the probationers, which was, he said, both theoretical and practical. One great point that they always insisted upon, was teaching the nurses the importance of their responsibility, and the value of punctuality and obedience to orders. It was said that every woman was a nurse. But this was far from the truth. A nurse required great physical strength, evenness of temper, an absence of bustle and noise, tact, and good management. An educated nurse had no theories of her own—she carried out her orders, and kept a record of each case for the observation of the doctor. The old-fashioned nurse would cry out, "Lah, sir, if I had not been here she would have gone off in a fit!" There was nothing of this in the trained nurse. Unobserved by the patient, she kept her record, and quietly showed it to the doctor on his arrival. Every hour she watched the progress of the case, but she was never nervous, frightened, or excited. The lecture was listened to with interest, not only by the nurses and medical officers of the institution, but by several ladies and district nurses from the town. After the lecture many of the visitors went through some of the wards, and made themselves practically acquainted with various nursing details.

MATERNITY AND DISPENSING CLASSES.—Dr. Griffith writes from the Hospital for Women and Children, 9 and 32, Lupus-street, S.W.: "As I am having so many applications from ladies about the Maternity and Dispensing Classes in connection with the hospital for which certificates are given (the certificates are recognised by all licensing bodies) would you, through the medium of your valuable paper, state that the classes are open to any students wishing to join.

NURSES AND LUNATICS.—With regard to trained attendants for the insane, the following quotation from Dr. Tuke's article on "Lunatics as Patients, not Prisoners," may be interesting to our readers: "What every case of insanity demands as the primary condition for recovery is separate and individual treatment and consideration. What every asylum requires in order to become a truly curative institution, is a hospital for the treatment of recent and acute cases, separate and distinct from the main establishment, to which each patient should be consigned on admission. Its medical staff should be ample, and their duties should be entirely restricted to the observation and treatment of new cases. The patients should be tended by properly trained nurses, not by ordinary attendants casually drawn from the servant and labouring classes. They should be submitted to the same systems of examination as patients in general hospitals. Every scientific appliance for the diagnosis and treatment of disease should be called into requisition, and every phenomenon should be carefully recorded. In a word, each patient should be treated on the purest hospital principle for at least a year, unless, of course, recovery has been reached in a shorter period." Dr. Tuke believes that such a system would lead to a rapid increase in the number of recoveries.

CONSECRATED WOMEN.—It is not many years since all trained nurses of any worth were members of religious sisterhoods, and in Catholic countries this fact still holds good. Every nurse ought to consecrate her life more or less to her work, because it is labour which demands the best that can be offered, and is not to be lightly taken up or hastily repented. But the days of convents, we hope, are over for ever, and women, with their great capacities for doing as well as for suffering, will never again in great numbers lock themselves up behind barred gratings. Some excitement has been manifested in Scotland because a nun took the veil at the Convent of Perpetual Adoration at Dumfries on April 11. This is the first time since the Reformation that such a ceremony has been performed in Caledonia, and the Prioress of the Order travelled all the way from Arras to present the novice. The ceremony of taking the veil is a suggestive one; the novice comes in dressed a bride, but shortly her gay garments are taken from her, black robes and black veil are thrown over her, and she prostrates herself before the altar. Then the priest reads the burial service over her, and she is dead to the world. Her future consists in the petty trivialities of the convent and in the perpetual adoration, which is one of its strictest rules. What a different life to that offered by nursing sisterhoods! To fly from the world is surely a cowardly act; to fight with evil as manifested in disease is surely a brave one. If women wish to consecrate their lives, let them consecrate them to deeds, and not to words.

### The Nursing and Management of the Insane.

By T. Duncan Greenlees, M.B. Edin.

*(Being introductory to a course of lectures to the Nurses of the City of London Lunatic Asylum.)*

*(Continued from page vii.)*

There are certain legal enactments to which I wish to direct your attention, as it is important that you should all be thoroughly acquainted with those laws affecting you as nurses in a public asylum. As I have already said, the law exercises a jealous care over all those certified to be insane, and should a nurse so far forget herself as to be cruel to a patient, or injure her in any way, the law punishes that nurse most severely. No amount of provocation from the patient will be accepted in extenuation of your crime, and you will receive no sympathy or protection from those in whose employment you are.

The following two clauses, obtained from the Lunacy Laws, show the gravity of wilful neglect or injuring of insane patients :

"1. Any attendant who in any way abuses, illtreats, or wilfully neglects a patient is liable to a fine of £20, or may be indicted for misdemeanour and punished by imprisonment.

2. Any attendant who, through wilful neglect, permits a patient to escape from an asylum, or abets or connives at the escape of a patient, for any such offence, he or she, shall incur a penalty of £20."

Every attendant or nurse on joining the staff of a lunatic asylum is made to sign some such obligation as follows :

"I hereby promise to obey the rules of this asylum, to faithfully execute the orders given me by my superior officers, and to perform any duty assigned to me, although not of a kind for which I am chiefly engaged. I consider myself bound to promote the objects of the institution, to do my best to further the recovery of the patients, and to secure their comfort and safety. I also undertake not to bring into the institution any intoxicating liquors, to be careful of its property, to avoid all gossip as to its inmates or affairs, and to endeavour generally by my conduct and demeanour to sustain its reputation. If anything improper is done in my presence or to my knowledge, I pledge myself to lose no time in reporting it to the medical superintendent or to one of the superior officers. I understand my engagement to be monthly, and agree to give one calendar month's notice should I wish to leave my situation. I acknowledge the right of the medical superintendent to discharge me without warning, and with forfeiture of all wages, for acts of harshness or violence to the patients, intemperance, or disobedience to orders."

My earnest advice to you is to live up to the character of your obligation, and, in your efforts to do so, you will have the sympathy of your companions, and the encouragement and co-operation of your superior officers.

The following excellent advice is given to nurses in a little handbook, written many years ago by Dr. Forbes Winslow, and now, I believe, out of print :

"Be kind, considerate, and courteous in your behaviour ; never resent anything done to you by a patient, but remember that persuasion and kindness are better than force and harsh words, and endeavour to make the patients respect you."

All men and all women cannot make good and successful attendants on the insane ; temperaments so differ among different people that there are really few who are naturally fitted to make good asylum attendants. In the first place, it is essential that an attendant should enjoy perfect physical health ; a healthy body gives a happy and contented mind, and illhealth is nearly always associated with an irritability of temper, not conducive to successful work.

There are certain qualifications of character necessary for success in your occupation ; among these I may mention tact and good temper. Some people possess tact, and an equable temper already as part of their natural disposition ; others have to acquire these qualities by experience and "through trials and tribulations." They can only be acquired by a careful and continuous watching of yourselves, and by exercising a self-control at all times. Any display of temper is fatal to good work ; it impairs your influence in the wards, and among your patients ; exposes you to the danger of their animosity, and, as all evil habits are contagious, it creates a general state of irritation both among the patients and your companion nurses, which is not seemly among the insane, and still less so among the sane. If self-control is difficult at first in a ward full of insane patients it will become in time easy enough, and will then smooth over many little troubles that would otherwise have proved almost insurmountable.

Tact is one of those qualities of character that is born with the individual, and cannot easily be acquired ; nevertheless, by careful exercise and observation, you may learn in time to be "all things to all men" in its best and noblest sense. The ability of managing your fellow-men is a quality to which you should studiously endeavour to attain, and if you do not already possess it avail yourselves of every opportunity of acquiring it. These opportunities you will find an abundance of in the wards.

There is no occupation that demands a larger share of patience and forbearance than the care of the insane. Your patients are irresponsible for their actions and conduct, and they may be obstinate, irritable, insulting, and even possess perverted habits and tastes ; all these abnormal characteristics—the symptoms of the disease from which they suffer—are to be met by patience and forbearance. Retaliation for any word or act of your patients will always be found to result in ill-feeling, and it may be in revengeful thoughts or desires against you. On the other hand, by exercising patience with and kindness to your patients, you will find that they will improve in their manner towards you ; and in time your good example will exercise a beneficial effect on all around you. By a continual and careful observance in your daily life of the qualities I have referred to, you will make yourselves better fitted for whatever sphere of life it may be your lot to be placed in ; you will make yourselves better women, and you will be loved by those under your care, and respected by your companions and superiors.

Obedience, discipline, punctuality, and neat habits among the staff are all essential to the success of any institution. In your dealings with your patients, remember that you are always an example to them. Their powers of imitation are great, and if you wish to succeed your conduct should be above reproach. Be kind to them, and have consideration for their failings, and strive to direct them into better and healthier channels, both of thought and action.

A kind and gentle spirit will support them if feeble, and soothe them if irritable and fractious.

> . . . . "For pity makes the world
> Soft to the weak, and noble to the strong."

Your patients have to be treated with candour and truthfulness ; do not treat their ailments lightly, for an apparent slight illness or pain is often the source of much discomfort to those whose troubles, real or imaginary, are as great burdens to bear as they are to those who are sane. There is such a thing as mental pain which, I believe, in certain cases, is more severe than any amount of physical pain. What may seem to us a ridiculous or foolish delusion is to them an intense reality ; and when you consider this perhaps your manner towards them will be tempered with greater kindness and gentleness.

*(To be continued.)*

### Nursing in the Soudan.

*(Continued from page vi.)*

### Part III.—In the Desert.

Picture to yourselves a low wooden building with walls some 7ft. high, and above them an open space of 5ft., and above that, again, the wooden roof supported by uprights, and you have the fabric glorified by the high-sounding name "The Auxiliary Hospital." The floor, the bare sand of the desert; tables of the roughest description—deal boards placed upon a tub or any other safe foundation ; low iron military bedsteads, and a chair or two—these made up what was really a fairly comfortable sick room. All was snug enough, unless perchance a sandstorm was raging ; and then woe betide everyone, whether an inmate of an open building or a more substantial stone edifice. I defy the most philosophical to feel comfortable under such circumstances ; the only thing is to endure, and when the storm has passed to set to work and shovel the sand off beds, tables, and chairs, making the best of your food afterwards.

The deft hands of the sisters had already put a very home-like look upon everything inside. Smart rows of bottles, basins, and jugs, instruments and utensils, polished and shining, and they themselves so cheery and bright—what more *can* a sick soldier want ? The bare boards of the walls were soon, as if by magic, adorned with pictures from the illustrated papers, while the soldiers were chatting of dangers passed and of homes far away in dear old England.

Now for the sisters' quarters, which were as primitive as the rest of their surroundings. A portion of the hospital building, some 14ft. or 15ft. square, with a little slip of a room divided off to form bath and dressing-room (for no one could exist there without a "tub"), constitutes their bed, sitting-room, and culinary apartment. To jump out of bed, or bath, on to the bare sand is, to say the least, amusing, provided there are no scorpions or lizards in close proximity, and, oh! the difficulty of taking that bath in retirement, or dressing in comfort, for remember that necessary but embarrassing 5ft. of open ventilation all round! What merry peals of laughter over the straits, difficulties, and dangers! But everything comes to an end at last. The dressing was accomplished, one sister goes off to the wards to take her share with the orderlies in the ward work, while her companion is lighting the stove and cooking the breakfast. It was suggested that the scorching sun might have performed that duty, but it didn't.

The medical officers were most kind and helpful, and every one vied with the other in doing their best to relieve and lessen pain, suffering, and discomfort. There were 100 beds in the hospital, but the number of patients varied. As the heat increased, so did the patients, and, of course, after an engagement many were brought in. Nursing in hot climates has some advantages, however; our experience was that wounds healed well. Medical diseases, principally fevers, were aggravated by the heat, and even when a patient was out of danger the great difficulty was to recoup lost strength, and many a one sank in the effort. The temperatures of the patients were most erratic; a man would come in at night with a temperature of 106deg., yet by the morning it was normal, while other symptoms were entirely wanting, the patient apparently being pretty well. The why and wherefore of all this is, of course, a question for the medical profession, but the fact is one of interest to nurses.

The question of food is another great difficulty in a hot climate. The Government supplies, aided by the invaluable National Aid Society's stores and provisions, left our patients little to complain of; but it must be borne in mind that there comes a time when Brand's essence and tinned milk begin to pall upon the fastidious appetite of a sick man. To go out and milk the cow or kill the ox was impossible, and fresh vegetables were things only to dream of. Add to all this the plague of flies and ants; one had but to expose one's food for one instant, and, as if by magic, it became black with those villainous insects. Thus you have some idea of the many difficulties that beset the work of nursing sick soldiers in the Soudan.

The hospital ship "Ganges" was a marvel of an orderly, well-regulated hospital. Immense credit was due to the medical officers and sisters for their admirable organisation, to say nothing of their kindness and attention to their sick and wounded patients. To the thoughtfulness of friends in England, and the Red Cross and National Aid Societies, the soldiers were indebted for many forms of recreation. In the hospital were to be seen almost every illustrated paper, newspapers, and books; and of games, there were chess, dominoes, draughts, cards, and solitaire, which the men much enjoyed when convalescent. Those who were strong and well were to be seen amusing themselves with cricket, football, and lawn tennis, incredible as it may seem, with the thermometer standing at anything between 80deg. and 90deg. in the shade. But so it was, and there is no accounting for tastes! The sisters had to thank the officers for many acts of kindness, as for instance, on one afternoon, when a lull occurred from nursing bad cases, following upon much hard work, it was proposed that those of the sisters who could be spared should visit the camp and see for themselves what life there really was during active service. Accordingly, with an escort consisting of two officers, a correspondent of an English daily paper, and the artist of an illustrated weekly, they set off through the town, and across the causeway to the mainland, where hundreds of white tents were pitched, passing *en route* long rows of horses and camels belonging to the Madras cavalry, picketed in the open. Those Indian soldiers were very picturesque in their white turbans and gorgeous apparel. Presently, after threading our way through this animated scene, we found ourselves in an officer's tent, where we were hospitably entertained at afternoon tea. It must be borne in mind that a soldier's tent in time of war has no luxuries to boast of, and only necessaries of the barest description. To find enough seats was a great difficulty: one camp stool, two old packing-cases, a biscuit tin, and the like were soon brought into requisition; but not sufficing for the entire party, the rest had to make themselves comfortable on the floor of the tent. This accomplished, a far greater difficulty presented itself—where were the cups and saucers? Empty Swiss condensed milk tins were found to be admirable substitutes, and these, with *one* cup and saucer, sufficed for most of the party; the others waited a second course. No tea ever tasted better, or was more thoroughly appreciated. We left our hospitable entertainers, laden with trophies captured from the poor Soudanese.

Sunday, March 29th, we heard rumours that a service would be held in a tent on Quarantine Island. Those of us who were able were anxious to attend. We could not, however, find any notice of the place or hour, so set off upon an exploring expedition from tent to tent. At last we were directed to a large one standing a little away from the rest. Upon entering we found it all but full of officers and men, whose grave faces and attentive looks spoke volumes; for all knew that on the morrow they were expected to engage in deadly conflict with the enemy, and who among that crowd could foresee what that morrow had in store for him? The chaplain, in a few kindly, well-chosen words, reminded them of this. Deeply touching was the scene as their strong voices, with evident emotion, sung the well-known words, "Jesu, Lover of my soul."

In the afternoon the "Iberia," with the Australian contingent, entered the harbour. Excitement ran high, and every effort was made to give the gallant fellows a hearty welcome. As soon as the huge troopship had come to anchor, cheer after cheer from those who lined the quay rent the air, answered back merrily and joyfully by the dense mass of Australians who crowded every available space on deck and rigging.

The natives, who swarmed around, stared with open-eyed amazement at the strange sight; curious thoughts must have passed through their minds of the wonderful scenes being enacted in their midst, as to what all the shouting meant, where those red-coated men had come from, and what a wonderful people those Britishers were. But now the gangway was lowered, high officials had gone on board, and were heartily shaking hands with those in command; arrangements were made, and the men disembarked and formed into column, to march into camp. Merry strains of music were heard, the Guards' band had come out to meet them and play them in. Fine big fellows those Australians were, and very proud and happy they looked as they marched along, the observed of all observers.

*(To be continued.)*

### Vaccination and its Opponents.

The *Bristol Medico-Chirurgical Journal* (March, 1889) reviewing some books on vaccination says:

"Vaccination has got into undeserved discredit by the way in which its details have been carried out by thoughtless or careless operators. It is much to be desired that all vaccinations should be taken out of the hands of private practitioners, and allowed to be performed only by public vaccinators. The difficulties in the way of this much-needed reform could be easily overcome. Vaccination, as an important branch of preventive medicine, should be under Government inspection. Not only is there great difficulty, privately, in obtaining trustworthy lymph, often necessitating a resort to unauthorised sources, but, in deference to the sentimental objections of ill-informed parents there are many practitioners of good social standing who are not ashamed, by vaccinating by one or two small insertions, to earn a cheap popularity, although thereby a serious danger is added to the life of a child thus made unfit to successfully resist a possible attack of smallpox. There are also doctors of a lower grade who set themselves up in unwholesome opposition to the Public Vaccinator, and, by performing the operation for a degrading fee of sixpence or a shilling, with a vaccination also much reduced in quantity—and therefore in quality—draw off a considerable number of ignorant mothers from the vaccination station, the efficiency of which becomes impaired through a greatly diminished attendance seriously limiting the selection of lymph, and the proper management of which becomes well-nigh impossible.

"In the light of Marson's figures (Seaton's 'Handbook of Vaccination,' ed. 1868, p. 216; McVail, p. 36; Woodward, p. 15), confirmed by all after-experience, conduct such as this, in various walks of professional life, seems little short of criminal, and has now reached such appalling magnitude as to urgently call for Government inteference.

"If vaccination is to be a reality, and not merely something which leads its subjects into a fools' paradise, the State must ensure, by an inspection through properly-qualified officials, that it is carried out in all ranks of society in a thoroughly efficient manner."

### "OFF DUTY" HOURS FOR NURSES.

Miss Louisa Twining has written a letter to "Nursing Notes" on the subject of nurses and their leisure hours. It appears that in many infirmaries the nurses' "off duty" time is in the evening, between seven and nine o'clock—surely a very unsuitable period of the day for a young woman's recreation. In summer it is pleasant enough, perhaps, but for nine months of the year it must be very much the reverse if the nurses spend their time in walking the streets. And it must be remembered that many public gardens are closed in the evening. No mother would send out her young daughters to amuse themselves at such an hour, and we cannot think that a matron is justified in permitting such recreation hours for young nurses. When infirmary nurses were mostly middle-aged, it might not matter so much; but now, when the majority of them are young women, it is distinctly unwise. And it is almost certain that they will not spend a couple of hours in a cold evening in walking up and down the streets. They go to some place of amusement, probably one they had better never have entered; and, even if it were the most harmless in the world, it prevents them getting the fresh air and exercise they are supposed to be seeking. Evening is the leisure time for the home circle, and perhaps for men. But women are not men—a fact which is sometimes forgotten by reformers, who require the warning of the wise Frenchman that "equality of attainment between men and women does not mean identity of nature." For women's outdoor amusement or recreation the afternoon is the best time, and we hope that ere long evening "off duty" hours for nurses will be very exceptional indeed, and granted only when the matron knows exactly how they are to be spent.

### Appointments.

[It is requested that successful candidates will send a copy of their applications and testimonials, with date of election, to The Editor, The Lodge, Porchester-square, W.]

City Hospital, Birmingham.—Miss Ambler-Jones has been appointed superintendent of nurses at the above hospital. She was trained at St. Bartholomew's Hospital, and has been for eighteen months superintendent of nurses at the London Northern Fever Hospital.

Salford Royal Hospital.—The following appointments have been made this week at the Salford Royal Hospital: Junior house surgeon (resident), Thomas Ashton Goodfellow, M.R.C.S. (Eng.), L.R.C.P. (Lond.). District medical officers (non-resident): Broughton district, Frederick G. Robinson, M.B. (Victoria), M.R.C.S. (Eng.), L.R.C.P. (Lond.); Salford district, Frederick Hyde Folkes, M.R.C.S. (Eng.), L.R.C.P., L.S.A.

### Everybody's Opinion.

*Correspondence on all subjects is invited. No communications can be entertained if the name and address of the correspondent is not given, or unless one side of the paper only be written on.*]

### LEICESTER INFIRMARY.

C. F. writes: Whenever I go for a holiday I make a point of visiting all the objects of interest in the neighbourhood, and, of course, to me a hospital of any kind is one of the most interesting objects in creation. So, when I was at Leicester the other day, having done my duty to the antiquities of the town—the Roman pavement; the bridge Richard III. rode over on his way to Bosworth; the Town Hall, where Shakespeare played before Queen Elizabeth - having visited all these, I dragged my hostess off to the Infirmary. The Leicester Infirmary stands in a good open space, but in near proximity to a large number of factories, so that it is sure to receive plenty of accident cases. At present, building operations are going on in connection with the new Children's Hospital, and one or two wards are closed for cleaning, so we didn't see everything, but we were charmed with what we did see. Even my companion, who had assumed that a hospital must be "so very depressing," was converted by the cheery wards, with their beautifully painted chimney-pieces—done by a sister—the piano in most, the screens hung with Indian muslin, and dainty ribbon-trimmed baskets for bandages. But what pleased me most was the admirable accommodation provided for the nurses. There was a piano in the sitting-room; the furniture, though not plentiful, was good; but the most charming of all were the nurses' bedrooms. Each had a whole room—not merely a cubicle—and though they were not large, they were delightfully light and airy, very much better than the apartments of the sisters in many of our London hospitals. Of course, individual taste showed in decoration, but thanks to Japan and the pampas, one can always make a den nice at little cost nowadays, and most of the rooms were exceedingly pretty. I believe that till recently the accommodation for nurses was very bad at Leicester, but now the committee has taken the matter up, and done it well. And yet they don't indulge in a debt at Leicester—seem to think it a superfluous luxury. I am not going to speak of the courtesy of Miss Rogers, the lady superintendent, and the charming nurse who showed us over the Institution, for—though some people do seem to fare otherwise—I never met with anything but courtesy in a hospital, whether or not I had any right there. But I did reflect that if the Fates were to bid me become a pro., I should ask them to send me to Leicester.

### PRIVATE INSTITUTIONS.

One That Likes Fair Play writes: It is very pleasant to see how much money is earned by the different institutes by private nurses; it would be far pleasanter to see more spent for the comfort of the nurses. The matron can have all her nights in bed and every comfort, while nurse has to earn the money, never a kind word from either committee, ladies, or gentlemen that would cheer her on in her cases. All the praise is given to the matron. No nurse is asked if she is comfortable, or if she has a complaint to make, but matron can say what she likes against a nurse to the committee. Nurse has no voice in any matter; she is only a machine or clock, made to go, fit or not fit. Is that a *nurses'* home?

### NURSING INSTITUTIONS.

Combination is sorry to read of "Contented Ones" having so little sympathy for suffering nurses. They cannot be aware of the ill-treatment of nurses in the commercial nursing institutions, or, as Christian women, they could not write such an article. "Contented Ones" are right in saying, "Nursing ought not to be looked at in the light of a money-making profession," and if they could only impress upon the proprietors of private nursing institutions how unholy it was to make their fortunes out of sorrow and sickness, and the labour of poor nurses, who risk their health and lives for their benefit, it would be an act of charity. About "Contented Ones" having plenty to eat and wear, it is well known that if they did not find plenty to eat and wear also for their so-called superiors, their services would not be required in a private nursing institution with a proprietary body

### Vacancies.

The following vacancies are announced:

### Lady Superintendents.

Northamptonshire Institution of Trained Nurses.
Bradford Infirmary, £80.

### Nurses.

Royal Infirmary, Bristol, one charge and two assistant nurses.

Royal Infirmary, Windsor, night, £20 to £25.

St. Cuthbert's Poorhouse, Edinburgh, two, £25.

Hospital for Incurables, Mauldeth, Manchester, £30.

Institution of Trained Nurses, Leicester, four.

Frome Nursing Institution.

Buckinghamshire Infimary.

Arbroath Infirmary.

Newport and County Infirmary.

Manchester Hospital for Incurables, £30.

Glasgow Maternity Hospital, £30.

Wimbledon Nursing Institution.

Carmarthenshire Infirmary, £20.

Eastbourne Nursing Institution.

Church Deaconess House, Chester, £24.

Kendal Hospital, £28.

Levick Institution, Paris.

### Probationers.

Royal Infirmary, Bristol (several).
Kent Nursing Institution.

